# Real-Time 3D High-Resolution Microscopy of Human Cells on the International Space Station

**DOI:** 10.3390/ijms20082033

**Published:** 2019-04-25

**Authors:** Cora Sandra Thiel, Svantje Tauber, Christian Seebacher, Martin Schropp, Rainer Uhl, Beatrice Lauber, Jennifer Polzer, Srujana Neelam, Ye Zhang, Oliver Ullrich

**Affiliations:** 1 Institute of Anatomy, Faculty of Medicine, University of Zurich, Winterthurerstrasse 190, 8057 Zurich, Switzerland; svantje.tauber@uzh.ch (S.T.); beatriceastrid.lauber@uzh.ch (B.L.); jennifer.polzer@uzh.ch (J.P.); 2 Department of Machine Design, Engineering Design and Product Development, Institute of Mechanical Engineering, Otto-von-Guericke-University Magdeburg, Universitätsplatz 2, 39106 Magdeburg, Germany; 3 TILL I.D. GmbH, Am Klopferspitz 19a, 82152 Martinsried, Germany; seebacher@till-id.com (C.S.); schropp@till-id.com (M.S.); rainer_uhl@me.com (R.U.); 4 National Aeronautics and Space Administration (NASA), ISS Utilization and Life Sciences Division, Kennedy Space Center, FL 32899, USA; neelamsrjn@gmail.com (S.N.); ye.zhang-1@nasa.gov (Y.Z.); 5 Ernst-Abbe-Hochschule (EAH) Jena, Department of Industrial Engineering, Carl-Zeiss-Promenade 2, 07745 Jena, Germany; 6 Zurich Center for Integrative Human Physiology (ZIHP), University of Zurich, Winterthurerstrasse 190, 8057 Zurich, Switzerland; 7 Space Life Sciences Laboratory (SLSL), Kennedy Space Center, 505 Odyssey Way, Exploration Park, FL 32953, USA

**Keywords:** high-resolution microscopy, structured illumination microscopy, live cell imaging, International Space Station, microgravity, immune cells, cytoskeleton, cell dynamics

## Abstract

Here we report the successful first operation of FLUMIAS-DEA, a miniaturized high-resolution 3D fluorescence microscope on the International Space Station (ISS) by imaging two scientific samples in a temperature-constant system, one sample with fixed cells and one sample with living human cells. The FLUMIAS-DEA microscope combines features of a high-resolution 3D fluorescence microscope based on structured illumination microscope (SIM) technology with hardware designs to meet the requirements of a space instrument. We successfully demonstrated that the FLUMIAS technology was able to acquire, transmit, and store high-resolution 3D fluorescence images from fixed and living cells, allowing quantitative and dynamic analysis of subcellular structures, e.g., the cytoskeleton. The capability of real-time analysis methods on ISS will dramatically extend our knowledge about the dynamics of cellular reactions and adaptations to the space environment, which is not only an option, but a requirement of evidence-based medical risk assessment, monitoring and countermeasure development for exploration class missions.

## 1. Introduction

Cellular and molecular reactions in response to altered gravity are often very fast and dynamic. In gene expression studies in human T cells and cells of the myelomonocytic system, response times of only 20 s were detected upon alterations of the gravitational force (hyper- and microgravity) [1,2,3], followed by rapid adaptation processes with 5 min [1,2,3]. The macrophageal oxidative burst reaction responded in the range of seconds [4,5] and adapted in less than 1 min [5].

Signal transduction cascades are often non-linear, highly dynamic [6], oscillating [7] and integrate multiple simultaneous signals within the structural complexity of a cell [8,9]. Thus, end-point-analysis approaches have only very limited evidence and in gravitational biology, we have just started to discriminate initial and primary effects from secondary reactions and adaptation processes induced by gravitational changes [1,3,10]. Due to limited technical and operational resources, gravitational biology research data are usually end-point-measurements, allowing no information about dynamic processes and therefore severely restricting the understanding of the dynamics of the processes, raising the danger of misinterpretation and error. It is very obvious, that investigation of cellular or molecular reaction and adaptation processes in altered gravity environments requires dynamic measurements and high-resolution live imaging technologies.

Confocal laser-scanning microscopy (CLSM) is used extensively in the scientific and industrial communities, particularly in the materials sciences and life sciences [11]. It was invented by Marvin Minsky [12,13], became very common and popular in the scientific community in the late 80 s [14], and has been recognised as a state-of the-art imaging method of living specimens for more than two decades [15,16,17,18]. The idea and concept for CLSM in microgravity research were studied for suborbital ballistic rockets and space applications very early [19] and was also recommended by the National Research Council’s (NRC) Committee for the Decadal Survey on Biological and Physical Sciences in Space [20].

A few custom-made microscope instruments have been built by research groups for parabolic flight experiments [21,22,23,24,25]. Since 2015, the FLUMIAS-TEXUS confocal laser spinning disk fluorescence microscope (Airbus DS) has been available for experiments on parabolic flights and suborbital rockets [26], allowing high-resolution images of living cells and cellular structures using four different lasers. Despite the enormous technological advances in microscopy in recent decades, no high-resolution fluorescence microscopy for live imaging experiments in different gravity conditions on board the ISS has thus far been available. The slow rotating centrifuge microscope NIZEMI (Zeiss Axioskop, Zeiss, Germany) has been successfully used during the IML-2 (International Microgravity Laboratory-2) mission on STS-65 in 1994 [27,28]. It was able to record transmitted light images of cells, but was not suitable for high-resolution imaging.

It took more than 25 years from the first conceptualization [19] to the first confocal capability was established on board the International Space Station (ISS). A confocal module with 532 nm frequency-doubled Nd:YAG laser was installed on the Light Microscopy Module (LMM) in the Fluids Integrated Rack (FIR) ISS facility, allowing 30 frames per second of confocal images. The first 3D images on the ISS were taken on 12 April 2018, from colloids of the Advanced Colloids Experiments (ACE) [29,30]. However, the single excitation line design limits its usage for life science applications.

**Figure 1:**
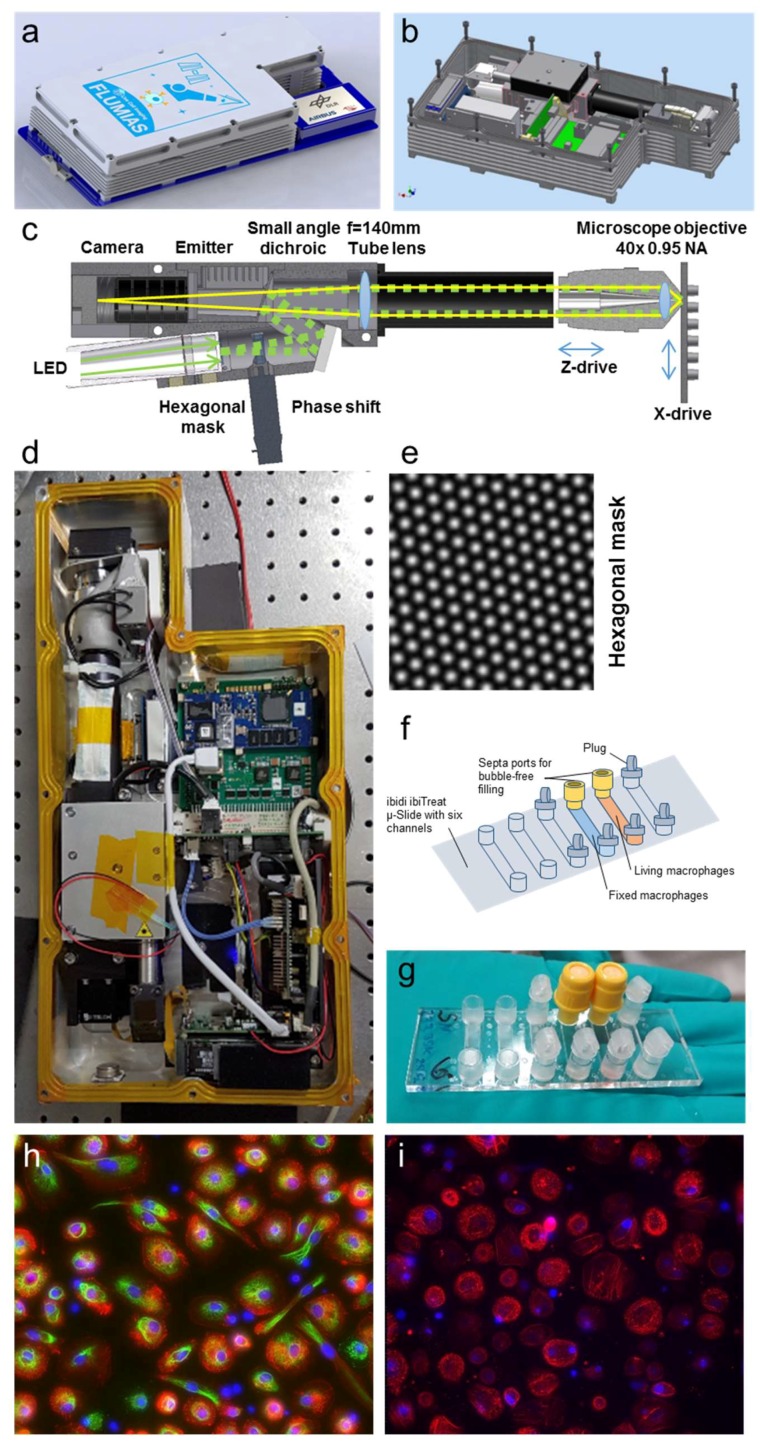
The FLUMIAS-DEA microscope. (**a**) CAD-image of the microscope attached to the Tango Lab carrier card. (**b**) CAD-image with the double sealed cover removed. (**c**) Optical beam path of the microscope. The excitation light coming from the LED is structured by passing the hexagonal mask and moved by the phase shift motor. A quad-band small angle dichroic is reflecting the excitation light towards the sample passing the tube lens and focused by the Nikon 40×, 0.95 NA air corrected objective. The objective is moved by a piezo to acquire z-stacks. The ibiTreat µ-Slide VI 1.9 (custom made by ibidi, Martinsried, Germany, channel volume app. 120 µL, referred to as ibidi µ-Slide) integrated into the sample holder can be moved on one axis to address two of the six ibidi µ-Slide channels. The fluorescence light passing the dichroic mirror is filtered by a quad band emitter and is detected by the camera. (**d**) Photograph of the microscope. (**e**) Intensity distribution of the structured illumination patterned excitation. (**f**) Scheme of cell culture vessel with living and fixed macrophages. (**g**) Photograph of ibidi µ-Slide with living and fixed macrophages. (**h**) Three channel overlay of maximum projected SIM-images of the fixed sample; size is 370 × 370 µm. Staining and imaging parameters of the fixed cells can be found in Table 2. (**i**) Two channel overlay of maximum projected SIM-images of the living macrophage sample; size is 370 × 370 µm. Staining and imaging parameters of the living cells can be found in Table 2.

The FLUMIAS-ISS microscope of the German Aerospace Center (DLR) is under development aiming to provide high-resolution 3D fluorescence live-cell imaging capability based on structured illumination microscopy (SIM) technology [31], with an integrated centrifuge systems allowing examination of numerous biomedical samples under various gravitational conditions on the ISS. SIM is a method to obtain confocal-like and (potentially) resolution-enhanced images by image processing of a set of raw images obtained after shifting the pattern between raw image acquisitions. We recently characterized the resolution limit of hexagonal pattern SIM [32], using commercial nanorulers, i.e., DNA origamis that consist of two distinct fluorescent labels with a fixed distance, as well as 50 nm diameter fluorescent microspheres and we were able to show a doubling of the resolution with our SIM system compared to a line confocal image [32] with both methods independently. Here we describe the first test of the FLUMIAS technology demonstrator (FLUMIAS-DEA) on the ISS, a simplified variant of the planned FLUMIAS-ISS microscope, which will be mounted on a centrifuge and will encompass auxiliary systems for cell cultivation. This first demonstration unit also employs a hexagonal mask [32] instead of a line grid or rectangular pattern [33]. The hexagonal intensity distribution in the sample is generated by imaging a hexagonal mask (Figure 1e) through a tube lens (TL) and the microscope objective onto the sample (see Figure 1c). In contrast to previous SIM-concepts, the hexagonal pattern merely needs to be shifted in a single direction, thus making pattern rotation obsolete, because the hexagonal mask contains spatial frequencies in at least three directions. This simplification permits a much more compact microscope-design, allowing to fit the whole 3D-microscope into a net-volume of <5 L.

The FLUMIAS-DEA microscope was designed and built by TILL I.D. and integrated in the Space Tango facility on ISS by Airbus DS on behalf of DLR. In our study, we tested the FLUMIAS technology and the operational processes, using two test samples: (1) Living primary human macrophages, stained with the intravital dyes Nuclear Violet (for the nucleus) and SiR-actin (for the actin cytoskeleton), and (2) fixed and stained cells (cell nuclei/DAPI, vimentin cytoskeleton/anti-vimentin, actin cytoskeleton/SiR-actin). On 3 July 2018, the first set of 3D images of living and fixed human cells were obtained by the FLUMIAS-DEA microscope on the ISS and transmitted to a ground station. The acquisitions lasted 11 days and the images were examined for high-resolution image quality and actin cytoskeleton dynamics.

## 2. Results

The FLUMIAS-DEA experiment was performed as a technology demonstration and as feasibility study for the planned high-resolution FLUMIAS-ISS microscope, which will be built and brought into service soon after the FLUMIAS-DEA microscope’s space capability has been successfully demonstrated. For the accommodation of the FLUMIAS-DEA microscope, only a volume of 7 L was available in the Space Tango facility on board the ISS. Another challenge was the very short development period of only 10 months from initial conceptualization until the flight to the ISS. For this reason, it was necessary to reduce the technical and experimental requirements to a minimum, which are described in the following section.

LED lighting was used instead of laser light sources allowing excitation wavelengths of 405, 475, 550, 640 nm. Bright field imaging was not included in the microscopic applications. A Nikon 40×, 0.95 NA air corrected objective together with an autofocus system was installed in the microscope. The stage displacement was possible in x and y direction. Observations were merely allowed in microgravity and not during the upload phase. Furthermore, a centrifuge to generate 1 g or partial gravity was not foreseen in the system. Subject of investigation were adherent non-proliferating cells seeded in a modified ibidi µ-Slide, allowing for on orbit analyses under a limited life support for at least 7 days. The cells had to survive during the entire experiment time in a total volume of 120 µL cell culture medium without medium exchange because pumps were not included in the system. Additionally, the chosen cell type had to be able to tolerate ambient temperatures during integration and pre-flight ground control measurements as well as temperatures of 25–27 °C during the upload to the ISS (packed in a phase shift material) and temperatures of 28–35 °C during the entire measurement time in the Space Tango facility (without active temperature control). Additionally, to the live cell sample, a fixed cell sample with three differently stained cell structures was included to test the full functionality of the instrument. The experiment was conducted automatically following a pre-defined program sequence. However, the option was included to upload program updates and to download pictures via the Space Tango facility interface. After each recording, compressed pictures (maximum intensity projections (MIPs)) and housekeeping data were downloaded. The number of possible recordings was determined by the memory capacity of the system of a 2 TB solid state drive (SSD) hard drive.

Figure 1 describes the FLUMIAS-DEA microscope technology. The microscope housing has a total volume of 7 L and fits into the Space Tango facility (Figure 1a,b,d). The interface connections are compatible with the Space Tango facility for controlling and data transfer. The excitation light coming from the LEDs transmits an intensity modulating mask, the central element of the SIM technology, resulting in a hexagonally patterned excitation intensity (Figure 1e). A quad-band small angle dichroic reflects the excitation light towards the sample (Figure 1c, right) passing the tube lens and focused by the 40×, 0.95 NA air corrected objective. The excitation intensity in the microscopic sample is moved by the phase shift unit. For the reconstruction of one SIM image, at least 7 raw frames have to be recorded. In order to increase redundancy and thus system stability, we decided to record totally 8 raw frames for each SIM image to process.

The objective is moved by a piezo to acquire the z-stacks. The sample can be moved in the x direction to address two adjacent samples. The fluorescence light from the sample that passes the dichroic mirror is filtered by a quad band emitter and is detected by the camera.

On the biological side, various parameters and the associated margins for live cell cultivation had to be tested before the start of the mission (Table 1). A maximum cultivation period of 87 days, with an optimum of 21–30 days, was determined for primary human macrophage cell culture. The optimal cell count for seeding in ibidi µ-Slides was tested as well as the optimal concentration for non-toxic long-term staining of the living dyes SiR-actin and Nuclear Violet (Table 1 and Table 2). Furthermore, we examined the margins for the cultivation time of the cells with SiR-actin and Nuclear Violet in the medium (Table 1). The parameters for staining of the fixed cells were also investigated (Table 2).

Table 3 summarizes the mission preparation procedures starting 10 months before the launch with the FLUMIAS-DEA design concept. Primary human macrophages were selected as test system for the demonstrator flight. Primary human macrophages were differentiated from monocytes, isolated from blood donations, and 25,000 to 35,000 cells were seeded in two channels of ibidi µ-Slide (Figure 1f,g). At differentiation day 14, the cells in one channel were fixed and stained with DAPI, anti-vimentin, and SiR-actin (Table 2, Figure 1h). The living cells in the second channel were stained with 50 nM SiR-actin and 1µM Nuclear Violet (Table 2, Figure 1i).

**Figure 2:**
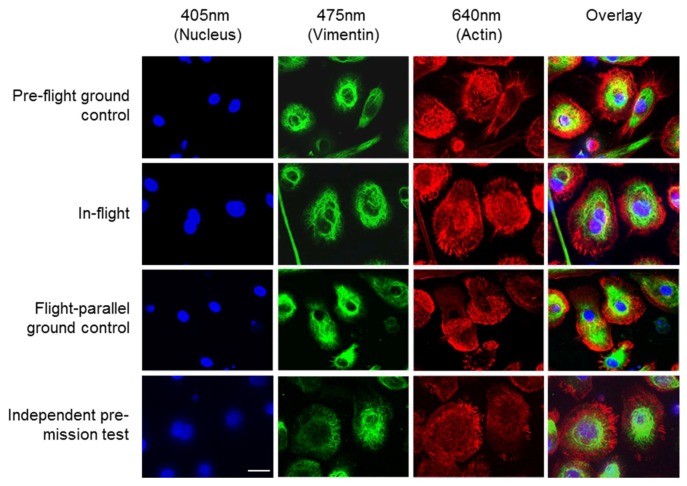
Fluorescence microscopy images of fixed primary human macrophages exposed to microgravity on the ISS. Cells were stained for nuclei (DAPI, blue), vimentin (anti-vimentin-Alexa Fluor 488; green) and actin (SiR-actin, red). Images were acquired on ground before upload to the ISS (Pre-flight ground control), and after 4 days of exposure to microgravity on the ISS (In-flight). Also shown are pictures of cells that were cultured and imaged parallel to the flown cells (Flight-parallel ground control) and images from a pre-test of the FLUMIAS-DEA microscope (Independent pre-mission test). The “Flight parallel ground control” images were acquired with a standard confocal microscope, while all other pictures were taken with the FLUMIAS-DEA microscope. Scale bar: 20 µm. Images show representative cells from the respective conditions.

**Figure 3:**
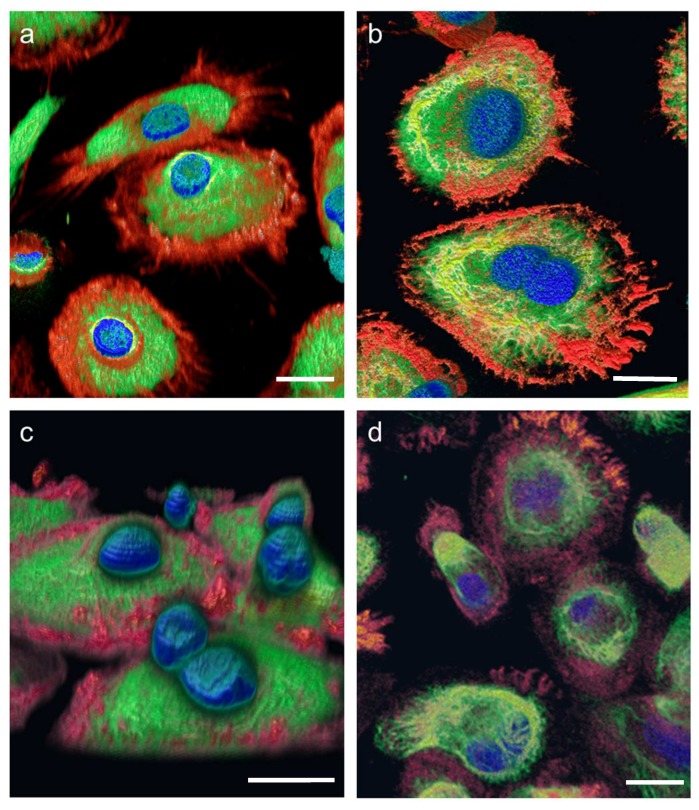
3D-reconstructions of fixed primary human macrophages exposed to microgravity on the International Space Station (ISS). Cells were stained for nuclei (DAPI, blue), vimentin (anti-vimentin-Alexa Fluor 488; green) and actin (SiR-actin, red). Fluorescence microscopy images were acquired (**a**) on ground before upload to the ISS (Pre-flight ground control) (**b**) after 4 days of exposure to microgravity on the ISS (In-flight) and (**c**) on ground in parallel to the In-flight samples (Flight-parallel ground control). Additionally shown are (**d**) images from a pre-mission test of the FLUMIAS-DEA microscope (Independent pre-mission test). Image shown in (**c**) was acquired with a standard confocal microscope (Nikon A1R) while for (**a**,**b**,**d**) images were acquired with the FLUMIAS-DEA microscope. Images show the localization of cell structures in 3D. Note: Scale bars are 20 µm.

**Figure 4:**
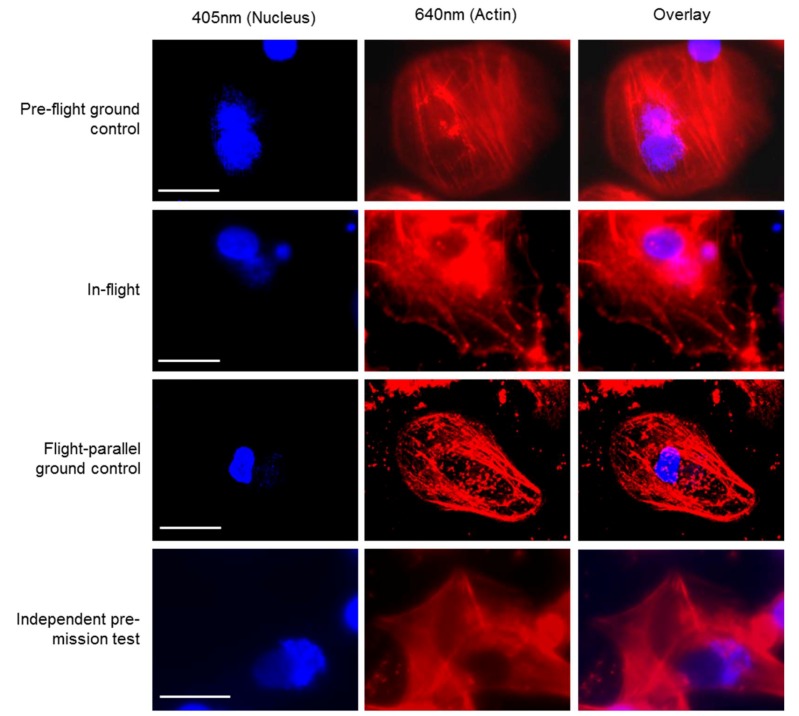
High-resolution fluorescence images of living primary human macrophages exposed to microgravity on the ISS. Cells were stained for nuclei (Nuclear Violet, blue) and actin (SiR-actin, red). Images were acquired on ground before upload to the ISS (Pre-flight ground control), and after 4 days of exposure to microgravity on the ISS (In-flight). Also shown are images from cells that were cultured in parallel to the in-flight samples (Flight-parallel ground control) as well as pictures of a pre-test of the FLUMIAS-DEA microscope (Independent pre-mission test). The “Flight-parallel ground control” group was acquired with a standard confocal microscope, while all other pictures were taken with the FLUMIAS-DEA microscope. Images show representative cells from the respective condition. Scale bars are 20 µm. For description of experiment sample groups see Table 4.

**Figure 5:**
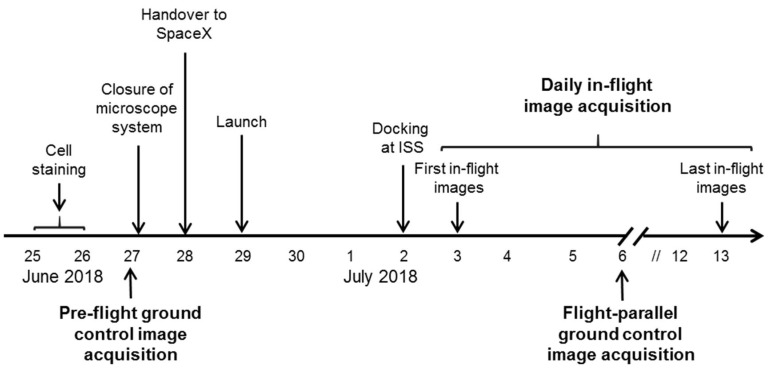
Experiment sequence of the FLUMIAS-DEA investigation aboard the ISS.

The ibidi µ-Slide was closed and integrated into the FLUMIAS-DEA microscope. Pre-flight ground control measurements of the fixed and living cells were performed (Figure 2, Figure 3 and Figure 4). The instrument was then handed over to the operator for installation in the Dragon capsule. On 29 June 2018 at 5.42 a.m. local time, it was launched with Space X CRS-15 from Cape Canaveral, FL, USA. 3 days later, the FLUMIAS-DEA microscope was implemented into the Space Tango Lab facility on board the ISS, the instrument was switched on and the pre-programmed acquisition settings were started automatically. Measurements were performed daily. The image acquisition sequence was to first record one stack of the fixed cells and then to record stacks of the living cells every 15 min over a period of 2 h (Figure 5, Table 3). Experiment groups were descripted in Table 4. After each measuring unit, overview images of the stacks were downloaded, the microscope was reset by switching off and on again and was thereby restarted. In-flight measurements were performed until 14 days after the launch. Simultaneously to the on orbit measurements, 7 days after launch, we performed flight-parallel ground control measurements with a Nikon A1R confocal microscope (Figure 2, Figure 3 and Figure 4, Table 4). At the end of the microscopic measurements on board the ISS, data packages were downloaded and the microscope was finally switched off 21 days after launch and prepared for the return to earth. After the landing of the Dragon capsule on 3 August 2018, the microscope and the solid state drive with the data were sent back and data processing of the SIM images as described previously [32,34] was started (Table 3).

Figure 2 shows images of the high-resolution acquisitions of the fixed cells on orbit and in three different ground controls. The nuclei (blue), the vimentin cytoskeleton (green) and the actin cytoskeleton (red) were stained (see Table 2). The cell nucleus, the individual structures, and fibers of the cytoskeleton can be detected in detail (Figure 2). Additionally, 3D volume reconstructions were calculated from the SIM images using the FLUMIAS-DEA. These 3D images show the exact localization of labelled structures within the cell (Figure 3). Additionally, the cell morphology as well as cellular structures can be viewed in Supplementary movies 1–3 representing experiment groups: “Pre-flight ground control”, “In-flight” and “Flight-parallel ground control”, respectively.

The FLUMIAS-DEA microscope worked properly on ground and in microgravity on orbit. Handling, transport and upload did not affect the technical components and high quality and high-resolution images were recorded (Figure 2 and Figure 3, Supplementary movies 1 and 2). Similar to the fixed cells, we analyzed living cells stained with Nuclear Violet (blue) and SiR-actin (red). Example pictures of the in-flight and ground control samples are shown in Figure 4. For the live cell imaging, the FLUMIAS-DEA microscope was fully functional and delivered high quality images on orbit and on ground (Figure 4).

**Figure 6:**
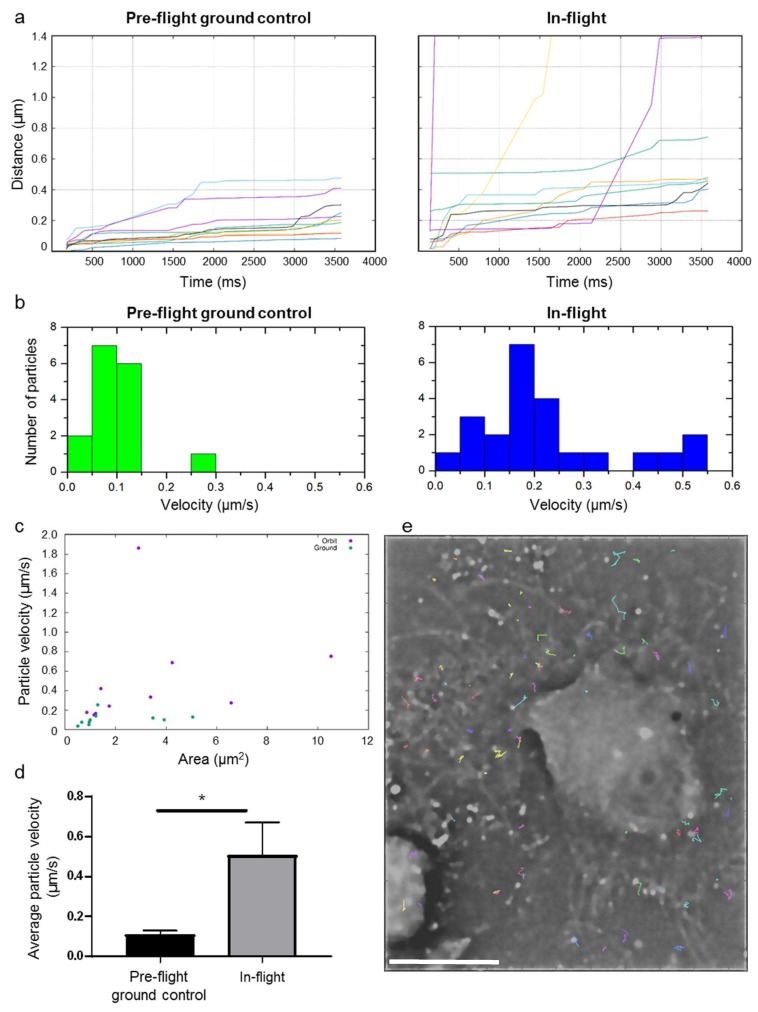
Comparison between cell dynamics In-flight and in Pre-flight ground control: (**a**) Particle movement path lengths of tracked particles from the samples In-flight and Pre-flight ground control. (**b**) Average particle velocities have been calculated from the path lengths, which are compared with histograms in-flight versus pre-flight ground control. (**c**) Area of the tracked particles versus velocity. Velocity was not dependent on particle size. (**d**) Comparison of average particle velocities in the Pre-flight ground control and In-flight. Error bars represent SEM. Statistical evaluation was performed with a t-test. *p*-values < 0.05 were considered as significant (* *p* ≤ 0.05) (**e**) Display of 76 trajectories of tracked particles on a picture of the F-actin-cytoskeleton recorded In-flight. Scale bar: 20 µm.

Figure 6 shows a first analysis of the cell dynamics of living cells in microgravity. The dynamics of the actin cytoskeleton was measured within a set of 24 raw images. In order to obtain better temporal resolution, the SIM pattern was removed from every raw image by a Fourier filter instead of standard SIM processing. Then, foci labelled with SiR-actin fluorophores (for simplicity reasons named particles) in the sample have been tracked in two time series of 24 pattern removed raw images, where one time series was recorded in-flight and the other pre-flight on ground. We analyzed in total 76 spots for tracking in the in-flight images, and 33 in the pre-flight ground control measurements.

We chose those foci, which could be tracked in all 24 images for further evaluation. This selection was performed in order to get rid of e.g., detected noise/hot pixels in the detected intensity spots. The next evaluation step was to determine the traveled path lengths in the sample over time from the tracked particle coordinates; the comparison of average path lengths in-flight versus pre-flight ground control is shown in Figure 6a. On average, the spots in the in-flight sample moved further than those in the pre-flight ground control measurements.

The average particle velocities in-flight is compared with those on ground in Figure 6b,d. The velocity of the fastest of the selected particles on ground was found to be 0.25 µm/s, where the fastest tracked particle in-flight moved with a velocity of 1.86 µm/s (for visualization reasons, the fastest particle in-flight in not shown in the histogram; Figure 6b, right). The average velocities for the selected particles in-flight were 0.50 µm/s and thus approximately five times higher than average velocities measured for pre-flight ground control, which amounted 0.11 µm/s (Figure 6d). This means that the measured particle velocity in human primary macrophages in microgravity increased approximate 5-fold compared to the particle velocity in pre-flight ground control sample. Figure 6c shows the areas of the tracked particles versus their average velocities, indicating that there is no correlation between particle sizes and average velocities. An example of tracked particle trajectories for the in-flight measurement is shown in Figure 6e.

## 3. Discussion

The mission objective of FLUMIAS-DEA was to test the technology and operational procedures for a miniaturized high-resolution fluorescence microscope on the International Space Station (ISS) by imaging two scientific samples in a temperature-constant system: One sample consisted of fixed cells and the other sample was living human cells. FLUMIAS-DEA was a precursor test mission for the more complex FLUMIAS-ISS microscope with the aim of establishing powerful high-resolution fluorescence microscopy on the ISS, which provides real-time 3D imaging of biological samples. We successfully demonstrated that the FLUMIAS technology was able to acquire, transmit and store high-resolution 3D fluorescence images from fixed and living cells, allowing quantitative and dynamic analyses of subcellular structures, e.g., the cytoskeleton.

Apart from a constant temperature in the range of 30–35 °C, no other life-support measures were planned in this demonstrator mission. This means that living cells were not be supplied with fresh medium for the entire duration of the mission. We therefore selected a cell system, primary human macrophages, non-proliferating differentiated cells with extraordinary temperature robustness and a long life span which had already been tested to fulfil all biological margin requirements during previous ISS missions [10]. In pre-mission experiments, primary human macrophages were kept in culture for up to 87 days with regular media exchange. Pre-mission tests demonstrated that primary human macrophages stained with SiR-actin survived up to 30 days with regular media exchange and up to 21 days without media exchange in the FLUMIAS-DEA flight hardware. The preparation of the biological samples was facilitated by using adjusted multi-channel µ-Slides for microscopy from ibidi. Nevertheless, it should be noted that the foil at the bottom of the slides became partially wavy due to the necessary filling technique, so that not all areas in field of vision were in the same focal plane.

**Figure 7:**
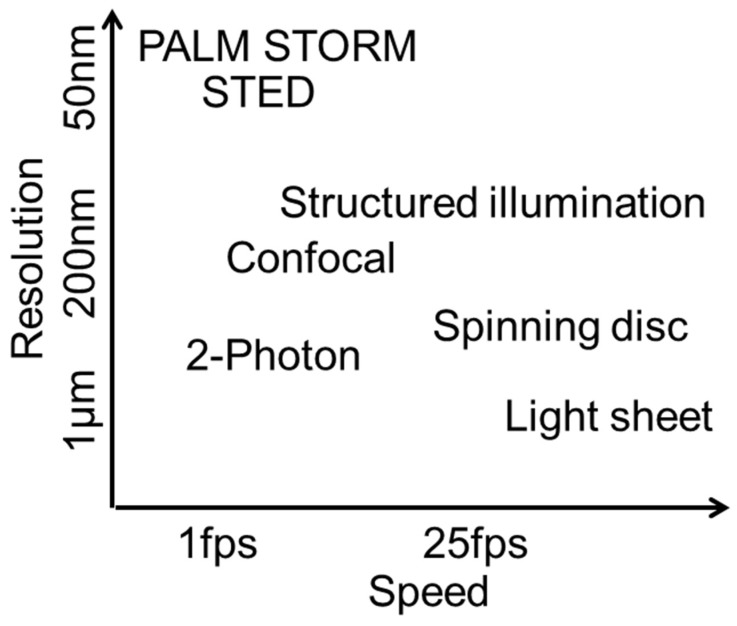
Classification of structured illumination microscopy (SIM) regarding resolution and imaging speed in comparison to other microscopy techniques. (fps: frames per second).

The FLUMIAS-DEA microscope combines several features of a high-resolution structured illumination microscope (SIM) with the requirements of a space instrument (Table 5, Figure 7). A normal high-resolution microscope system typically weighs more than 100 kg. The FLUMIAS-TEXUS confocal spinning disk microscope applicable on parabolic flights and suborbital rocket flights, for example, weighs about 120 kg. Intriguingly, the weight of the FLUMIAS-DEA was reduced to 6.5 kg (2.7 kg without containment). Some structural measures were necessary, such as the installation of LED light sources instead of lasers and the exclusion of emission filter exchanger. With its light weight, volume of only 7 L and a size of 400 × 200 × 90 mm, it is no bigger than a “shoe box”. In comparison to confocal microscopy, the “work horse” in scientific microscopy, structured illumination microscopy (SIM) increases the resolution in all three dimensions by up to a factor of approximately 1.4 and enables optical sectioning without the need for a pinhole [35]. Using a fine hexagonal grid pattern for illumination, SIM enables the detection of sample information by unmixing spatial frequency information from the microscopic object, which are unresolvable with other systems (Figure S1). SIM offers different possibilities for various data analyses. Super-fast cellular reactions can be monitored by investigating the raw images recorded for one image plane as a time lapse. These images are recorded within a time resolution of approximately 100 ms. Furthermore, 3D projections are possible, including all image planes of one stack. And finally, consecutive measurements over hours and days can deliver long-term information about cellular dynamics and adaptation processes. The SIM allows maximal flexibility for data evaluation and image processing for a wide range of research. As an example, after data acquisition, high-resolution processing can be chosen for maximum spatial resolution. In order to obtain (quasi-) confocal images, a much faster image processing can be selected. If a better temporal resolution is required (cell movements within the acquisition time of 8 SIM raw images), the pattern can be removed from the raw images for maximum temporal resolution (without spatial resolution improvement and confocality).

The versatile application is an important feature of a space research instrument, since many different research teams will use the instrument for different purposes. A compact, robust, lightweight, portable, highly sensitive fluorescence microscope would not only serve the research demand in space exploration, but also on the ground for use in remote areas, disaster conditions, environmental monitoring, chemical, food and beverage industries, and also as biological warfare agent detection for military and homeland defense [37]. The possibly of real-time analysis methods on ISS would dramatically extend our knowledge about the dynamics of cellular reactions and adaptations to the space environment, which is not only an option, but a requirement of evidence-based medical risk assessment, monitoring and countermeasure development for exploration class missions.

## 4. Materials and Methods

### 4.1. FLUMIAS-DEA Structured Illumination Microscopy (SIM) Fluorescence Microscope

The FLUMIAS-DEA microscope (Figure 1) is a modified version of a hexagonal structured illumination microscope (SIM) recently published in [32]. Instead of line confocal laser illumination, it uses a four color LED combiner and a global shutter camera MX124MG-SY-X2G2 (Ximea, Münster, Germany). The four LEDs are filtered with an excitation filter with passbands at 532–563 nm (Alluxa, Santa Rosa, CA, USA) and 389–408 nm, 461–485 nm, 636–644 nm (Semrock, Rochester, NY, USA). In the intermediate image plane of the excitation, a hexagonal mask with a pitch of 28.6 µm was placed. A 5 mm thick glass window mounted on a 6220 H galvanometer (Cambridge Technology, Bedford, MA, USA) was used to shift the excitation pattern. For each color eight shifts are done for each plane resulting in 8 phase shift images (raw images). The dichroic mirror is a custom ultra flat quad band filter with 18° angle of incidence matching to the emitter filter (Both Alluxa, Santa Rosa, CA, USA). A custom tube lens with 140 mm focal length was used to minimize the size of the instrument and optimize the sampling of the 3.45 µm pixels of the camera. A 40×, 0.95 NA objective MRD00405 (Nikon, European Headquaters, Amsterdam, Netherland) was used to image the sample. A Jetson TX2 Computer (Nvidia, Santa Clara, CA, USA) stored the raw images on a 2 TByte M.2 SSD 960PRO (Samsung, Seoul, Korea). A real-time computer based on Arm cpu and FPGA controlled the experiment. Focusing was done by a Piezo PZ400 (Piezo Jena, Jena, Germany) and an EDGE-4× piezomotor (Nanomotion, Yokneam, Israel) was used to move the sample in one direction up to 13 mm. The images were calculated offline after the flight similar to [32]. The complete microscope was placed in a 400 × 200 × 90 mm sized double sealed containment.

### 4.2. Isolation of Monocytes

Human monocytes were isolated from anonymized buffy coats, a by-product of blood donations, received from the blood transfusion service (Zurich, Switzerland; internal project registration no. 579) following a standardized protocol. Sterile 1× PBS (Biochrom GmbH, Berlin, Germany) was used to dilute buffy coats 1:2. Thereafter, 15 mL of the solution was layered cautiously on top of 10 mL Ficoll Paque Premium (GE Healthcare Bio-Sciences, Uppsala, Sweden) and subsequently centrifuged at 400 g for 30 min without break at RT. The layer of PBMC’s appearing at the interphase was transferred in a fresh sterile tube, and sterile 1× PBS (Biochrom GmbH, Berlin, Germany) was added to a final volume of 50 mL, then a second centrifugation step followed at 400 g for 10 min at RT. The supernatant was discarded, the remaining cell pellet was resuspended in 5 mL 1× PBS (Biochrom GmbH, Berlin, Germany) and 45 mL 1× PBS was added before another centrifugation step at 350 g for 10 min at RT. The washing step was repeated twice. The cell pellet was resuspended in 20 mL mononuclear cell medium (MCM) (Promocell, Heidelberg, Germany) and layered carefully on 25 mL of a 46% Percoll solution (10.64 mL Percoll, GE Healthcare Bio-Sciences, Uppsala, Sweden), 0.86 mL 10× PBS (Sigma-Aldrich Chemie GmbH, Steinheim, Germany), 13.5 mL RPMI (Biochrom GmbH, Berlin, Germany). Samples were centrifuged at 550 g for 30 min at RT without break. Monocytes that accumulated at the interphase were harvested, converted to a fresh sterile tube, 1× PBS was added to a final volume of 50 mL and the cell suspension was centrifuged at 400 g for 10 min at RT. In case the supernatant was still turbid, the washing step was repeated. The ultimate cell pellet of monocytes was resuspended in 8 mL MCM (Promocell, Heidelberg, Germany) and living cells were counted in a Neubauer chamber. For cell freezing, 22.5 million cells per aliquot were prepared and centrifuged at 350 g for 10 min at RT. Resulting cell pellets were resuspended in 1.5 mL cryo serum free medium (Promocell, Heidelberg, Germany), pipetted in 2 mL cryo vials and subsequently frozen at −80 °C. Vials were transferred to −150 °C for final storage one day after freezing. Frozen monocytes were transported from the home laboratory to the Space Life Sciences Laboratory (SLSL) at the Kennedy Space Center Florida using a temperature controlled (−196 °C) and GPS tracked transport container (Cryoport; Irvine, CA, USA).

### 4.3. Differentiation of Primary Human Macrophages

At the Space Life Sciences Lab (SLSL) (KSC, Exploration Park, FL, USA), the frozen monocytes were differentiated into primary human M1 macrophages following the standard protocol provided by Promocell (Heidelberg, Germany). For this purpose, cryovials with frozen monocytes (volume 1.5 mL) were thawed by slight but constant movement in a water bath at 37 °C for 2 min. The cell suspension was instantly transferred into 20 mL of MCM (Promocell, Heidelberg, Germany), which was pre-equilibrated for 30 min in a CO_2_ incubator at 37 °C. After incubation for 8–16 h (37 °C, 5% CO_2_, 95% humidity), cells in MCM were transferred in a sterile tube and centrifuged at 350 g for 10 min at RT. The cell pellet was resuspended in 3 mL M1 Macrophage Generation Medium DXF (Promocell, Heidelberg, Germany). Living cells were counted in a Neubauer chamber. The cell concentration was adjusted to 1.2 million cells per mL with M1 Macrophage Generation Medium DXF. 3.6 million cells (3 mL) of this suspension were transferred into each well of an 8 well plate (Thermo Fisher Scientific, Rochester, NY, USA). Alternatively, 25,000 to 35,000 cells were seeded directly into two of the six the channels of an ibiTreat µ-Slide VI 1.9 (custom made by ibidi, Martinsried, Germany, channel volume approximately 120 µL), referred to as ibidi µ-Slide, that had be degassing for 48 h at 37 °C. Cells in the ibidi µ-Slide were incubated for 6 days (37 °C, 5% CO_2_, 95% humidity). On day 6, a partial exchange of the medium was performed by exchanging 50% of the M1 Macrophage Generation Medium DXF in each well. A complete medium exchange was performed on day 9. The medium from each well, including cells in suspension, was collected in a sterile tube. 0.5 mL of fresh M1 Macrophage Generation Medium DXF was added immediately to the adherent cells in the 8 well plate to prevent desiccation of the cells. The cells in suspension were centrifuged at 350 g for 15 min at RT. The cell pellet was resuspended in 3 mL of fresh M1 Macrophage Generation Medium DXF and transferred back to its original well. Macrophages were ready-to-use for the experiments from day 10 on.

### 4.4. Experiment Preparation Protocol and Mission Scenario

#### 4.4.1. Staining of Primary Human Macrophages

25,000–35,000 primary human macrophages were seeded into one ibidi µ-Slide channel (see section “Differentiation of primary human macrophages”). Cells in one channel were fixed and stained, cells in a second channel were stained live before pre-flight imaging. Fixation and staining of fixed cells started 20 h before acquisition of ground control images (61 h prior to launch): Rinsing volume of the ibidi µ-Slide channel was 150 µL if not stated differently. Cells were fixed by rinsing the channel three times with 2% PFA (Sigma/Merck KGaA, Darmstadt, Germany) and incubated for 30 min at 37 °C in a humid chamber. Subsequently, channels were rinsed three times with PBS (Biochrom, Berlin, Germany) for washing. For permeabilization, channels were rinsed three times with 0.1% Saponin (Sigma/Merck KGaA, Darmstadt, Germany) followed by 15 min incubation at 37 °C in a humid chamber. Cells were washed as described above. For blocking, channels were rinsed three times with 3% BSA (Sigma/Merck KGaA, Darmstadt, Germany), followed by 1 h incubation at 37 °C in a humid chamber. Anti-Vimentin antibody (ab195877, Abcam, Cambridge, UK), labelled with AlexaFluor488 was added (3 rinses of 100 µL) in a 1:100 dilution, followed by incubation at 37 °C in a humid chamber for 2 h. After washing (as described above), channels were rinsed three times with 1 uM SiR-actin (Spirochrome AG, Stein-am-Rhein, Switzerland) for actin labelling. Cells were washed as described above and nuclei were stained by rinsing three times with 2 µg/mL DAPI (BioVision Inc., San Francisco, CA, USA) followed by 15 min incubation at 37 °C in a humid chamber. Cells were washed a last time as described above. Staining of living cells started 24 h before ground control images (65 h prior to launch): Channels were rinsed three times with 150 µL of medium containing 50 nM SiR- actin (Spirochrome AG, Stein-am-Rhein, Switzerland) and the ibidi µ-Slide was transferred back into the cell culture incubator. 12 h later (12 h before acquisition of the ground control images, 53 h prior to launch) the channel was rinsed three times with 150 µL medium containing 50 nM SiR-actin (Spirochrome AG, Stein-am-Rhein, Switzerland), 1 µM Nuclear Violet (AAT Bioquest, Sunnyvale, CA, USA), and additional 20 mM HEPES (Sigma/Merck KGaA, Darmstadt, Germany). As a last step the channels including channel ports were filled completely with the medium/dye mixture.

#### 4.4.2. Sample Preparation for Flight

After cells had been stained and shortly before the integration of the samples into the FLUMIAS microscope, the cell-containing channels of the ibidi µ-Slide were sealed (see Figure 1): The ibidi µ-Slide was positioned on a 37 °C pre-warmed metal block. Blotting paper was positioned between the sample ports to absorb excess fluid during the procedure. Aim of the procedure was to close the channels so that no air remained in the channels or in the channel ports. The channel with the fixed cells including the channel ports were filled completely with PBS (channel with the living cells including channel ports was already completely filled with medium/dye). To close one side of each cell-containing channel, septum-containing IN–Stoppers with luer lock connections (74.4312 Sarstedt, Nuembrecht, Germany, trimmed for fitting), were used. The “IN–Stoppers” were filled with the respective fluid of the channel and closed using a syringe with attached cannula. Subsequently, the cannula with attached syringe was pierced through the septum and the “IN-Stopper” was pressed onto the channel port. The syringe was then removed. The remaining cannula allowed for pressure equilibration while the other side of the channel was now closed with a fluid-filled hollow plug (luer plug male, 10822, ibidi, Martinsried, Germany). The two empty channels next to the cell-containing channels (see Figure 1) were closed on both sides with empty hollow plugs. As a last step the cannulas and the blotting paper with excess fluid were removed and the ibidi µ-Slide was ready to be installed into the FLUMIAS-microscope.

#### 4.4.3. Space Tango Facility on Board the ISS

The company Space Tango offers amongst other services, automated systems, so called TangoLab™ facilities, which allow to run and operate scientific payloads in CubeLabs™ modules on board the ISS. These facilities represent standardized platforms that enable to operate multiple biomedical and technology applications independently on orbit. An interaction with the payload from Earth is possible via the TangoLab facilities interface and downlink of data and images can be accomplished in near real time. Furthermore, the concept of the TangoLab facilities minimize astronaut interaction and saves thereby precious crew time. FLUMIAS-DEA was installed into the TangoLab facility for experiment execution on board the ISS.4.4.4. FLUMIAS-DEA image processing.

After the download and return of the FLUMIAS-DEA microscope and the transfer of the data, the SIM images were processed as described in [32,34]. Three dimensional volume reconstructions have been rendered by the software Amira 3D Software for Life Sciences from the company FEI (Thermo Fisher Scientific, Hillsboro, OR, USA).

### 4.5. Evaluation of Living Cell Dynamics

In order to analyze the dynamics of the living cell sample, we first chose a subset of 24 raw images. Then the frequencies of the structured illumination were determined with subpixel accuracy and the pattern was removed from the raw images by applying a fourier filter. In the next step, images were contrast enhanced by a Richardson-Lucy iterative deconvolution with total variation regularization [38]. We therefore analyzed a 3.7 s long time series with 3 blocks of 8 frames. The 8 frames had a time distance of 105 ms and a distance to the next block of 740 ms. After that a particle tracking software [39] was used to detect certain SiR-actin labelled spots in the images (see Figure 6e) and to track the detected spots in the set of 24 images. Subsequently, the travelled path lengths and velocities were evaluated for each tracked spot that could be identified in all 24 analyzed images (see Figure 6a,b) for a comparison of in-flight and pre-flight ground dynamics of the living sample.

## Figures and Tables

**Table 1:** Parameters and margins that were tested prior to the experiment. n/a: not applicable.

Parameter	Min	Max	Optimum
Cultivation time of primary human macrophages	n/a	87 days	14–30 days
Cultivation time of primary human macrophages when stained with SiR-actin and regularly re-stained together with medium exchange *	n/a	37 days	14–16 days
Cell number per ibidi channel	10,000	50,000	25,000–35,000
Cultivation of primary human macrophages in hardware when stained with SiR-actin without further medium exchange	1 day	21 days	14 days
Cultivation of primary human macrophages in hardware when stained with SiR-actin and Nuclear Violet without further medium exchange	1 day	14 days	1–5 days
Temperature range for cell culture	28 °C	37 °C	35–37 °C
Temperature range for upload	20 °C (max 24 h)	37 °C	28 °C
Non-toxic but effective dye concentrations for staining of live cells			
SiR-actin	20 nM	100 nM	50 nM
Nuclear Violet	0.1 µM	5 µM	1 µM

* SiR-actin staining starts at differentiation day 10.

**Table 2:** Sample staining and imaging parameters.

Sample Type	Staining	Concentration	Excitation Wavelength	Exposure Time
**Fixed cells**				
	DAPI	2 µg/mL	405 nm	50 ms
	Anti-vimentin	1:100	475 nm	130 ms
	SiR-actin	1 µM	640 nm	50 ms
**Live cells**				
	Nuclear Violet	50 nM	405 nm	50 ms
	SiR-actin	1 µM	640 nm	50 ms

**Table 3:** Sample preparation procedure for a (live) cell microscopy-experiment on board the ISS using the FLUMIAS-DEA microscope. L: launch

Time before Launch	Biology	Microscopy	Comment
L-10 months	Testing of biological parameters	FLUMIAS-DEA design concept	
L-6 months	Preparation of frozen cell stocks	Pre-mission preparation ongoing	
L-11 weeks	Science verification test	Science verification test	Mission-like test of biology in instrument
L-6 weeks	Transport of frozen cell stocks to launch site (SLSL)	Pre-mission preparation ongoing	Temperature controlled (−196 °C) and GPS-tracked (Cryoport; Irvine, California, USA)
L-17 days	Thawing and seeding of cells for microscope test	Pre-mission preparation ongoing	
L-14 to 15 days	Thawing and seeding of cells for flight	Pre-mission preparation ongoing	
L-9 days	Preparation of ibidi µ-Slide for microscope test	Pre-mission preparation ongoing	
L-7 days		Microscope transport to launch site (SLSL)	
L-7 days	Staining of live and fixed samples for microscope test	On-site preparation	Details for cell staining see Table 2
L-5 days		Microscope test	Details for exposure times see Table 2
L-65 to 53 h	Staining of live and fixed samples for flight	On-site preparation ongoing	
L-41 h		Final integration of sample and Pre-flight ground measurement	
L-38 h		Handover of closed microscope to Space Tango	Space Tango: Interface to ISS, operator of Space Tango facility
L-24 h		Package into stowing container and handover to SpaceX	
Launch 0	Space X CRS-15 Launch	Space X CRS-15 Launch; Ascent with a specific phase change material	29 June 2018, 5.42 a.m., Space X CRS-15
Launch +3 days		Implementation into Space Tango Lab facility; Evaluation of the housekeeping data; Automatic experiment run (28–35 °C)	
Launch +4 to 14 days		Microscopic measurements of fixed and live cells	
Launch +21 days		Power off microscope	
After landing		Sample removal, data transfer, transport of microscope to payload developer; Data processing and analysis	

**Table 4:** Experiment groups.

Experiment Group	Description
Pre-flight ground control	Ground control measurements of the flight samples after sample integration into the FLUMIAS-DEA microscope before launch
In-flight	FLUMIAS-DEA measurements on board the ISS
Flight-parallel ground control	Ground control measurements parallel to the FLUMIAS-DEA in-flight measurements on L+7 days with a Nikon A1R confocal microscope
Independent pre-mission test	Flight scenario like measurements were performed on 5 consecutive days during a pre-mission science verification test; shown are measurements on day 4 and 5

**Table 5:** Comparison of features and characteristics of structured illumination microscopy (SIM) with commercially available microscopy systems. Values in italics are estimates. n/a = not applicable. NA = numerical aperture

	Microscopy Systems			
Parameter	FLUMIAS-DEA	Confocal	Spinning Disk	Epifluorescence
Weight without table	2.7 kg without containment	80 kg	100 kg	25 kg
Max Power consumption	54 W	700 W	1000 W	250 W
Objective	40×/NA 0.95 air	40×/NA 0.95 air	40×/NA 0.95 air	40×/NA 0.95 air
Light source	4× LED	Multi laser	Multi Laser	Arc lamp /LED
Power at sample	5 mW	1 mW	10 mW	10 mW
Photon Detection Efficiency	70%	30%	50%	70%
Resolution xy FWHM (theory)	0.3 µm	0.3 µm	0.3 µm	0.3 µm
Super resolution xy	230 nm	none	none	none
Confocality z FWHM [36]	1.6 µm	1.7 µm	1.7 µm	none
Image size	3008 × 3008	2000 × 2000	1024 × 1024	2048 × 2048
Max 3D Pixel rate	45 MHz	1 MHz	20 MHz	none
Min 3D plane speed full field	200 ms	1 s	typical >50 ms	none
Min Epifluorescence speed	25 ms (raw image)	n/a	10 ms	10 ms
Max Epifluorescence Pixel rate	360 MHz	n/a	400 MHz	400 MHz
autofocus	Yes	possible	possible	possible

## Supplementary Materials

Supplementary materials can be found at https://www.mdpi.com/1422-0067/20/8/2033/s1.

## Abbreviations

CLSM	Confocal laser-scanning microscopy
DLR	German Aerospace Center
FWHM	Full width at half maximum
ISS	International Space Station
MIP	Maximum Intensity Projection
n/a	Not applicable
SEM	Standard Error of the Mean
SIM	Structured Illumination Microscopy
SLSL	Space Life Science Laboratory
tbd	To be determined
TL	Tube lens
