# Supplementary material for: Real-Time 3D High-Resolution Microscopy of Human Cells on the International Space Station
Source: Int J Mol Sci. 2019 Apr 25;20(8):2033. doi: 10.3390/ijms20082033 (PMC6514950; doi:10.3390/ijms20082033)
Supplement: Supplementary file 1 [file ijms-20-02033-s001.zip › ijms-485970-supplementary.pdf]

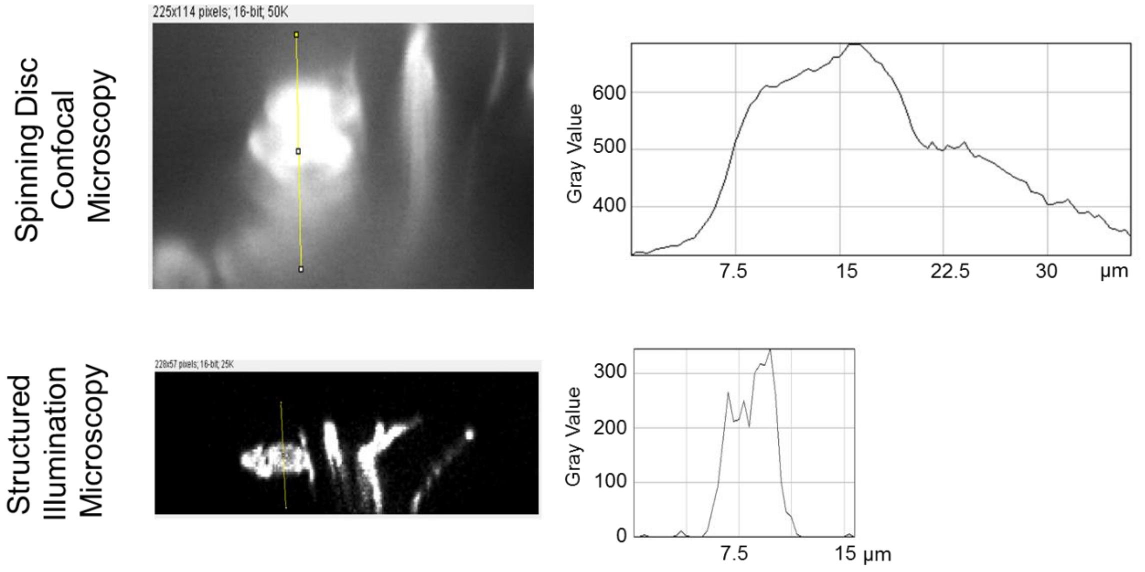

Supplementary Figure 1. SIM technology compared to spinning disk confocal technology. In the SIM technology, structures are better resolved, the fluorescence signal peak is more pronounced and the background signal is reduced leading to a better illustration of the investigated structures. Subject of investigation: convallaria majalis, Objective: 40x0.95 NA Air, Excitation: 488nm, Voxelsize: (x/y) 162nm, (z) 375nm (250nm mechanical distance combinedwith refraction index difference air/sample)

Supplementary movie 1: "Pre-flight_ground_control_3D_movie". 3D reconstruction of fixed primary human macrophages. The cell sample that was prepared for the measurements on board the ISS was imaged before the upload (pre-flight ground control). Cells were stained against nuclei with DAPI (blue), against vimentin with an antibody (green) and against the F-actin cytoskeleton with SiR-actin (red). Pictures were taken with the FLUMIAS-DEA microscope directly before the upload to the ISS. Details of staining and image acquisition can been found in the manuscript Table 2.

Supplementary movie 2: "In-flight_3D_movie". 3D reconstruction of fixed primary human macrophages that were exposed to microgravity on board the ISS. Cells were stained against nuclei with DAPI (blue), against vimentin with an antibody (green) and against the F-actin cytoskeleton with SiR-actin (red). Pictures were taken with the FLUMIAS-DEA microscope on board the ISS (In-flight). Details of staining and image acquisition can been found in the manuscript Table 2.

Supplementary movie 3: "Flight_parallel_ground_control_3D_movie". 3D reconstruction of fixed primary human macrophages. Cells were stained against nuclei with DAPI (blue), against vimentin with an antibody (green) and against the F-actin cytoskeleton with SiR-actin (red). Pictures were taken during ground control measurements that were performed parallel to the FLUMIAS-DEA in-flight measurements on L+7 days with a Nikon A1R confocal microscope. Details of staining and image acquisition can been found in the manuscript Table 2.
